# Well-Dispersed Cellulose Nanofiber in Low Density Polyethylene Nanocomposite by Liquid-Assisted Extrusion

**DOI:** 10.3390/polym12040927

**Published:** 2020-04-17

**Authors:** Tengku Arisyah Tengku Yasim-Anuar, Hidayah Ariffin, Mohd Nor Faiz Norrrahim, Mohd Ali Hassan, Yoshito Andou, Takayuki Tsukegi, Haruo Nishida

**Affiliations:** 1 Faculty of Biotechnology and Biomolecular Sciences, Universiti Putra Malaysia, UPM Serdang 43400, Selangor, Malaysia; tengkuarisyah@upm.edu.my (T.A.T.Y.-A.); faiznorrrahim@gmail.com (M.N.F.N.); alihas@upm.edu.my (M.A.H.); 2 Laboratory of Biopolymer and Derivatives, Institute of Tropical Forestry and Forest Products, Universiti Putra Malaysia, UPM Serdang 43400, Selangor, Malaysia; 3 Research Center for Chemical Defence, Universiti Pertahanan Nasional Malaysia, Kem Perdana Sungai Besi, Kuala Lumpur 57000, Malaysia; 4 Graduate School of Life Science and Systems Engineering, Kyushu Institute of Technology, 2-4 Hibikino, Wakamatsu-ku, Kitakyushu, Fukuoka 808-0196, Japan; yando@life.kyutech.ac.jp (Y.A.); nishida@lsse.kyutech.ac.jp (H.N.); 5 Innovative Composite Materials Research and Development Center (ICC), Kanazawa Institute of Technology, Hakusan, Ishikawa 924-0838, Japan; tsukegi@neptune.kanazawa-it.ac.jp

**Keywords:** cellulose nanofiber, nanocomposite, extrusion, melt-blending, liquid feeding, mechanical properties

## Abstract

Two different liquid assisted processing methods: internal melt-blending (IMB) and twin-screw extrusion (TWS) were performed to fabricate polyethylene (PE)/cellulose nanofiber (CNF) nanocomposites. The nanocomposites consisted maleic anhydride-grafted PE (PE*g*MA) as a compatibilizer, with PE/PE*g*MA/CNF ratio of 97/3/0.5–5 (wt./wt./wt.), respectively. Morphological analysis exhibited that CNF was well-dispersed in nanocomposites prepared by liquid-assisted TWS. Meanwhile, a randomly oriented and agglomerated CNF was observed in the nanocomposites prepared by liquid-assisted IMB. The nanocomposites obtained from liquid-assisted TWS exhibited the best mechanical properties at 3 wt.% CNF addition with an increment in flexural strength by almost 139%, higher than that of liquid-assisted IMB. Results from this study indicated that liquid feeding of CNF assisted the homogenous dispersion of CNF in PE matrix, and the mechanical properties of the nanocomposites were affected by compounding method due to the CNF dispersion and alignment.

## 1. Introduction

Cellulose nanofiber (CNF) derived from plant receives vast attention from researchers and industries due to its superior physical properties such as high crystallinity, thermally stable, biodegradable, biocompatible and non-toxic [1,2,3,4]. Owing to its superior properties, many studies have been conducted to determine CNF potentials in various field ranging from household materials to high-tech industrial applications [5]. CNF is a promising renewable nanomaterial that can replace the use of non-renewable materials in numerous products manufacturing, mainly as a reinforcement material for the production of nanocomposite [6,7,8].

Extensive studies on the use of CNF as the reinforcement material in polymer matrix have been carried out. Nevertheless, the hydrophilic nature and low thermal stability of CNF limit the choice of polymer matrices and processing technologies for CNF-based composite production [7,9]. Conventionally, CNF which is usually produced as a liquid suspension is dried prior to composite processing. This causes CNF to aggregate and poorly dispersed in the polymer matrix due to the formation of hydrogen bonds between the CNF during drying process [10]. In fact, the irreversible aggregation phenomenon of CNF upon drying leads to the size increment, and causing the nanofiber to lose its nano-scale characteristic [11]. This eventually contributes to the challenging process for the production of biocomposites reinforced dried-CNF.

Soulestin et al. [12] discovered that introduction of water injection during extrusion of low density polyethylene (LDPE)/cellulose fiber composites helped in improving fiber dispersion as well as eliminated cellulose aggregation. It was proven that water acted as a lubricant, thus reduced the effect of compounding process that could lead to cellulose degradation. Another method was also discovered by Oksman and colleagues [9], and it involves an introduction of nanomaterials in liquid form. It was revealed that liquid feeding of nanomaterials including CNF is beneficial in term of the followings: (a) unnecessary surface modification of CNF, (b) no degradation of surface modifiers, (c) low health risk because the CNF is in slurry and (d) CNF dispersion can be improved due to ‘blow-up’ phenomena, caused by evaporation of water from the CNF during melt-processing. This technique involves dispersing the nanomaterials in a liquid medium followed by feeding the suspension along with polymer during compounding [13]. This method has been applied to biopolymer-based composites such as polylactic acid (PLA), polyhydroxalkanoates (PHA) and polyhydroxybutyrate (PHB) [10]. To date, there is a limited report on the application of liquid feeding technique for polyolefins such as polyethylene (PE) and polypropylene (PP).

Apart from the CNF condition (dry/liquid), the mechanical properties of the final nanocomposites is also dependent on the compounding methods [14]. Different compounding methods may give different effect on the dispersion and orientation of CNF in the polymer matrix. As for microfiber, it is well understood that fillers or fibers will be randomly dispersed and poorly aligned within the polymer matrix during the internal melt-blending processing [15]. Meanwhile, an extrusion process produces well-dispersed and well-aligned micro-scale fibers in the matrix [16,17]. In the case of nanoscale fiber, the reinforcement effect might be different from the microfiber. It has been reported that nanomaterial creates percolating network within polymer matrix and hence it provides a strong reinforcement effect even at low fiber loading [18,19,20]. Percolating network is less likely to be formed when there is too little fiber or when the fibers are not well-dispersed [19] and hence at these conditions, nanocomposites tend to have reduced mechanical properties. To date, it is unknown if compounding methods of nanocomposites would affect its mechanical properties since the mechanism of reinforcement is different.

This study was therefore attempted to investigate the effect of liquid assisted processing and to clarify the effect of compounding methods on the morphological and mechanical properties of polyethylene (PE)/CNF nanocomposites.

## 2. Materials and Methods

### 2.1. Materials

Oil palm mesocarp fiber (OPMF) was supplied by Seri Ulu Langat Palm Oil Mill, (Dengkil, Selangor, Malaysia) and used as a starting material for cellulose nanofibers production. Low density polyethylene (LDPE, MFI 0.33 g/10 min, *ρ* = 0.92 g·mL^−1^), was supplied by Titan Petchem (M) Sdn. Bhd (Johor, Malaysia) and used as a matrix. Maleic anhydride-grafted-polyethylene (PE*g*MA, *ρ* = 0.92 g·mL^−1^) as a compatibilizer was purchased from SIGMA-Aldrich (St. Louis, MO, USA). Other chemicals, potassium hydroxide (KOH) and sodium chlorite (NaClO_2_) were also purchased from SIGMA-Aldrich (St. Louis, MO, USA) and used as received.

### 2.2. Methods

#### 2.2.1. Cellulose Extraction and Nanofibrillation Process

Cellulose extraction process was carried out as described in our previous study [21], with the aim to remove lignin, hemicellulose and other extractives. The obtained cellulose fiber was then dispersed in distilled water with a concentration of 0.2% (w/v) under vigorous stirring at 60 rpm for 24 h [22]. Cellulose suspension was then passed through a high-pressure homogenizer C50 (Avestin Inc., Ottawa, Canada) for 30 passes with an operating pressure of 50 MPa to prepare a 0.2 wt.% CNF dispersed solution. The resulting CNF solution was then stored at 4 °C prior to use.

#### 2.2.2. Preparation of Nanocomposites

**Figure 1:**
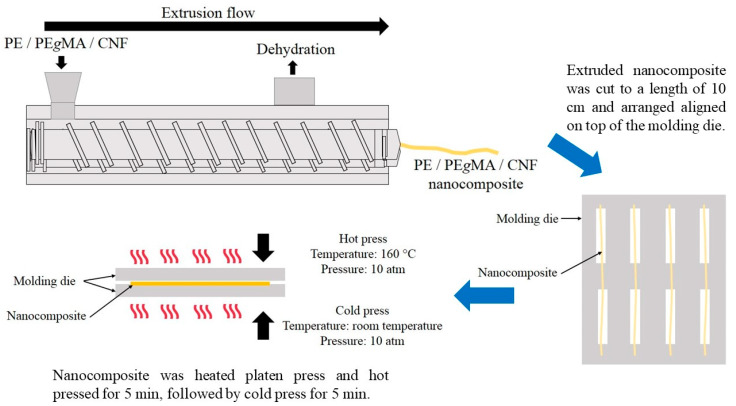
Schematic diagram of nanocomposite prepared by liquid-assisted twin screw extrusion.

Nanocomposites PE/PE*g*MA/CNF (97/3/0.5–5 wt./wt./wt.) were prepared using two different liquid assisted processing methods, which were twin screw extrusion (TWS) and internal melt-blending (IMB). The liquid-assisted TWS was conducted by a twin screw extruder (Model: IMC-160B, Imoto Machinery Co., Ltd., Kyoto, Japan) as shown in Figure 1. The liquid-assisted TWS was carried out at 50 rpm of a screw rotational speed, with a heating temperature profile of 80, 160, 160 and 160 °C, respectively for four zones of cylinder from hopper to die. Prescribed amounts of 0.2% (w/v) CNF dispersed solution, PE*g*MA as a dispersant, and PE as matrix were fed into the extruder cylinder and the mixture was melt-kneaded by shearing stress with proper rotation of screw, resulting in extrusion of PE/CNF nanocomposite as a strand from the die. The strand was cut to a length of approximately 10 cm and was then used for film preparation.

**Figure 2:**
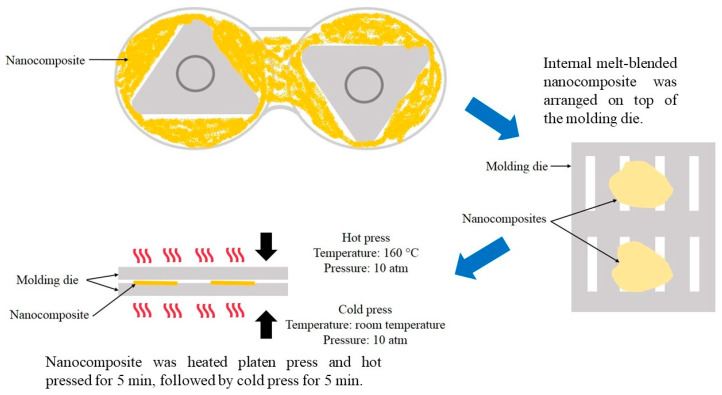
Schematic diagram of nanocomposite prepared by liquid-assisted internal melt-blending.

Liquid-assisted IMB process was conducted using a Brabender plastograph EC internal mixer (Brabender GmbH & Co. KG, Duisburg, Germany) with a mixing speed of 50 rpm for 20 min at 160 °C as shown in Figure 2. Prescribed amounts of PE, PE*g*MA and CNF dispersed solution were melt-blended and kneaded for 20 min.

The PE/PE*g*MA/CNF composites obtained from the two processing methods were placed on a molding die as described by Miyake and Imaeda [23]. For the composite prepared by twin screw extrusion, the sample was arranged in one direction, while the composite prepared by internal melt-blending was arranged randomly on the mold, and both samples were hot-pressed at 160 °C under 10 atm for 5 min to obtain film specimens for following analyses (modified from Then et al. [15]). All composites were prepared in prescribed weight ratios of LDPE, PE*g*MA and CNF (97/3/0.5, 1, 2, 3, 4 and 5 (wt./wt./wt.)) as shown in Table 1.

#### 2.2.3. Morphological Analysis

CNF dispersion in the nanocomposites was analyzed by examining the distribution of oxygen element using a scanning electron microscope equipped with energy dispersive spectroscopy (SEM-EDS) (JCM 6000, JEOL Ltd., Tokyo, Japan). Nanocomposite samples were firstly coated with platinum using a vacuum sputter coater prior to external morphology observation by SEM-EDS. While for internal morphology scanning of nanocomposites, an X-ray computed tomography device (X-CT) (Xradia 410 Versa, Zeiss, Oberkochen, Germany) was used at 20 kV with tube current 200 µA, scan time 10 s and scanning repetition of 1600 times exposure. Obtained X-CT images were then reconstructed using VGStudio MAX 2.2 (Volume Graphics GmbH, Heidelberg, Germany) to observe a cross-sectional image of nanocomposite samples.

#### 2.2.4. X-ray Diffraction Analysis

Crystallinity index (*CrI*) of nanocomposites was analyzed from X-ray diffraction (XRD) profiles measured with an X-ray powder diffractometer (Rigaku Corporation, Tokyo, Japan) equipped with nickel filtered Cu Kα radiation (λ = 0.1542 nm). The X-ray pattern was recorded in a 2θ range of 5–50° at 40 kV and 25 mA as well as at a scan rate of 2° min^−1^ [20,21]. The *CrI* was calculated based on Equation (1)

(1) $$C_{r}I=\left[\left(I_{002}-I_{\mathrm{am}}\right)/I_{002}\right]\times 100\%$$

where, *I*_002_ was diffraction peak intensity from (002) plain at 2θ = 23°, which corresponds to a specific diffraction peak of the crystalline portion. *I*_am_ refers to the intensity at about 2θ = 18°, and it refers to the amorphous region [21].

#### 2.2.5. Thermal Analysis

Thermal analysis of nanocomposites was conducted using thermogravimetry (TG) and differential scanning calorimetry (DSC) analyzers to identify decomposition and melting temperatures. Samples for both analyses were weighted in a range of 10–12 mg, and each sample was placed in an aluminum pan. TG analysis was performed using a thermogravimetric analyzer EXSTAR TG/DTA 6200 (Seiko Instruments Inc., Chiba, Japan) in a temperature range of 50 to 550 °C at a heating rate of 10 °C/min under a nitrogen flow of 20 mL/min [21]. DSC analysis was performed on an EXSTAR DSC 6220 (Seiko Instruments Inc., Chiba, Japan). In a first scan, the samples were heated from 30 to 200 °C at a rate of 10 °C/min and held at 200 °C for 1 min. They were then cooled to 50 °C at a rate of 10 °C/min and held at 50 °C for 1 min. Next for the second scan, they were again heated to 200 °C at a rate of 10 °C/min and held at 200 °C for 1 min. The thermal properties of samples were determined based on the data obtained during the second heating/cooling scan. Melting temperature (*T*_m_) was taken as the highest endothermic peak in the heating scan and cold crystallization temperature (*T*_c_) was determined based on the exothermal peak during the cooling scan [21].

#### 2.2.6. Mechanical Analysis

Tensile and flexural tests of the nanocomposite specimens were conducted using a compact tensile and compression tester IMC-18E0 (Imoto Machinery Co., Ltd., Kyoto, Japan). The tensile properties consisted of tensile strength, Young’s modulus, toughness and elongation at break were evaluated from stress-strain curves measured at a crosshead speed of 5 mm/min [24]. The flexural test was performed according to ASTM D790 on rectangular standard samples with dimension size of 120 mm × 12.7 mm and 3 mm thickness. Flexural tests, flexural strength and modulus were determined from stress-strain curves, which were measured at 10 mm/min [25]. The reported tensile and flexural properties values were averaged values of five measurements for each sample.

#### 2.2.7. Statistical Analysis

The mechanical properties of each nanocomposite were statistically analyzed using a one-way ANOVA in a general linear model using Statistical Analysis Software (SAS) Ver. 9.4. The mean for each measured parameter was separated statistically using Duncan Multiple Range Test (DMRT) at *p* < 0.05.

## 3. Results 

### 3.1. Morphological Analysis of Nanocomposite

**Figure 3:**
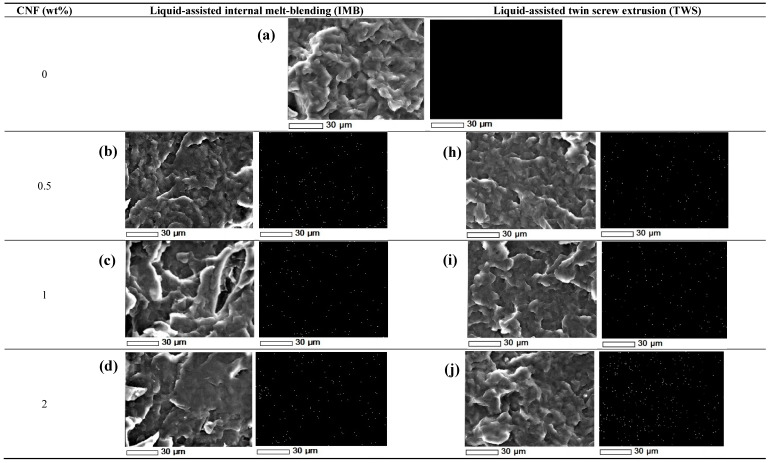

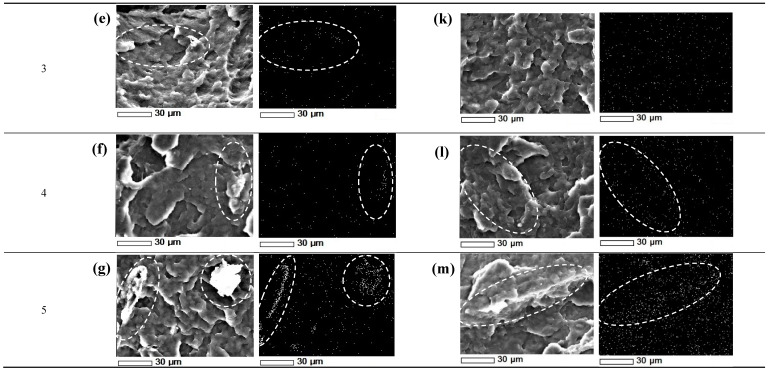
SEM images mapped with EDS analysis for distribution of oxygen element which represents CNF. (**a**) PE/PE*g*MA, (**b**–**g**) PE/PE*g*MA/CNF prepared by liquid-assisted internal melt-blending (IMB), and (**h**–**m**) PE/PEgMA/CNF prepared by liquid-assisted twin screw extrusion (TWS). Circles show agglomeration of CNF.

Figure 3 shows SEM and EDS images for the fractured surfaces of all samples. The observation of PE/PE*g*MA (Figure 3a) and PE/PE*g*MA/CNF nanocomposite (Figure 3b–g,h–m)) indicates differences across the surfaces among PE/PE*g*MA and PE/PE*g*MA/CNF nanocomposites. Figure 3a shows a smooth surface of PE/PE*g*MA and the thorough homogeneity of surface with no solid impurity proves that PE and PE*g*MA were remarkably compatible with each other.

From the micrographs in Figure 3b–g for nanocomposites prepared by liquid-assisted IMB, it was observed that PE/PE*g*MA/CNF nanocomposites with different CNF compositions exhibited different CNF dispersion states. As indicated in Figure 3b,c, the fibers distribution in the composites with 0.5 wt.% and 1 wt.% CNF prepared by liquid-assisted IMB seems to be homogenous. EDS images also showed the homogenous dispersion of white spots indicating the presence of oxygen, derived from CNF and PE*g*MA. The homogenous dispersion of CNF suggests a good wetting behavior between the CNF surface and PE/PE*g*MA matrix.

It can be observed that CNF was agglomerated, and homogenous dispersion was failed to be achieved when more than 1 wt.% CNF was incorporated in the PE matrix, as presented in Figure 3d–g. Interaction between CNFs happens due to surface forces to produce agglomerates, which are difficult to be re-nanofibrillated during the liquid-assisted IMB fabrication since the added shearing stress was not enough to break the agglomerates. This is supported by the results of Cruz and Viana [26] that the input process during melt-blending couldn’t break all the aggregate fillers, thus resulting in poor fillers dispersion. Herrera et al. [10] also reported that a high amount of CNF suspension may result in boiling and lead to fast evaporation of water, which consequently creating agglomeration of the CNF.

As for PE/PE*g*MA/CNF nanocomposites prepared by the liquid-assisted TWS, the homogenous dispersion of CNF was observed in the nanocomposites reinforced with 0.5, 1, 2 and 3 wt.% CNF as shown in Figure 3h–k. No agglomeration of CNF was detected from their EDS images. This proves that CNF can be dispersed homogeneously by the liquid-assisted TWS. Heterogeneous dispersion, however, was observed in the nanocomposites reinforced with 4 and 5 wt.% CNF as shown in Figure 3l,m, in which the SEM-EDS images showed unevenly distributed white spots, supporting the aggregation of CNF. Based on these results, the homogenous dispersion of higher amount of CNF was achieved by the liquid-assisted TWS, since the shearing stress during the processing must be enough to suppress and/or re-nanofibrillate the CNF aggregation. The presence of the second vent site at the end of the extruder also helps in preventing fast evaporation during composite processing and this reduces the potential of CNF agglomeration in the PE matrix.

Based on the overall morphological analysis, it was found that liquid feeding favored the CNF dispersion and distribution, as agglomeration was only observed in the PE/PEgMA nanocomposites consisted of 4–5% CNF for liquid-assisted TWS, and 2–5% CNF for liquid-assisted IMB. A similar report was also reported by Lo Re et al. [27]. They discovered that the liquid feeding can prevent agglomeration of fibers, as this method can prevent the formation of strong fiber-fiber interactions. Based on their findings, the fibers were found to be well-distributed in the PCL matrix for composites prepared by liquid feeding, whereas for composites prepared by dry feeding method, the fibers were found to be agglomerated and poorly distributed in the PCL matrix. Additionally, this method can also prevent the initial agglomeration caused by drying prior to compounding.

### 3.2. Mechanical Properties of Nanocomposite

The increment in mechanical properties was observed when CNF was incorporated in the PE matrix by both compounding methods as shown in Table 2. It was discovered that PE/PE*g*MA/CNF nanocomposites prepared by liquid-assisted TWS exhibited higher tensile strength, Young’s modulus, flexural strength and flexural modulus compared to PE/PE*g*MA/CNF nanocomposites prepared by liquid-assisted IMB. This outcome was highly related to compounding methods. Thus, this suggests that the existential state of CNF in the polymer matrix significantly reflected in the mechanical properties.

For nanocomposites prepared by liquid-assisted IMB method, the maximum mechanical increment was recorded when 1 wt.% CNF was incorporated in the PE matrix, as the values of tensile strength, Young’s modulus, flexural strength, and flexural modulus increased to 10.5, 145, 11.3 and 230.1 MPa, respectively. When the CNF composition exceeded 1 wt.%, the mechanical properties decreased along with the increase in CNF composition. Nevertheless, at 2 and 3 wt.% of CNF, the values were higher than those of PE/PE*g*MA as reference. A similar trend was also recorded for the nanocomposites prepared by liquid-assisted TWS processing. Increment in mechanical properties was observed along with the increase in CNF composition from 0.5 to 3 wt.%, reaching the highest mechanical properties at 12.1, 166, 16.5 and 273 MPa for tensile strength, Young’s modulus, flexural strength, and flexural modulus, respectively. It was also noticeable that by reinforcing with 3 wt.% CNF by liquid-assisted TWS, the mechanical properties of nanocomposites were considerably improved by more than 50% higher than PE/PE*g*MA.

In general, mechanical properties of composite increases by increasing the fiber loading in the matrix [28,29]. However, monotonous reduction in the overall mechanical properties was recorded when CNF composition was over 1 and 3 wt.% for liquid-assisted IMB and TWS, respectively. According to Mittal et al. [30], the reduction in mechanical properties occur after reaching a threshold limit and this is mainly due to poor mechanical interlocking, which may degrade load transfer between fibers and matrix. In addition to fiber loading, it could be seen from this study that the reduction in mechanical properties was also highly related to the agglomeration of CNF in the PE matrix during processing. As observed in the SEM-EDS images, agglomeration was observed when CNF composition was more than 1 and 3 wt.% for liquid-assisted IMB and TWS, respectively (Figure 3). This explains the reduction in mechanical properties. CNF agglomeration may also reduce the formation of a percolating network of CNF within the polymer matrix, hence reduce the mechanical properties of nanocomposites [7,19,31].

Findings from this study also confirmed that liquid feeding promoted better dispersion of CNF in the polymer matrix which contributed to improved mechanical properties of PE/PE*g*MA/CNF nanocomposites. These findings are in accordance with the findings from other studies as tabulated in Table 3. According to Lo Re [27], liquid feeding provided milder shear stress during compounding, thus the CNF length and L/D ratio can be preserved and indirectly resulting in better mechanical properties of composites.

Homogenous dispersion of CNF observed in SEM-EDS images is due to the usage of PE*g*MA as a compatibilizer, resulting in effective adherence between CNF surface and matrix PE. The good dispersion of CNF and wetting situation at the interface may also generate the desired stress transfer between PE/PE*g*MA matrix and CNF throughout the stress loading, resulting in excellent tensile and flexural strength of the nanocomposite. This has been reported by Essabir et al. [35], in which good compatibility between CNF and polymer matrix resulted in better adhesion and provided better stress transfer. Due to the reactive interaction of hydrogen bonding between hydroxyl groups on CNF surface and MA moieties in PE*g*MA, CNF is able to be bound with PE*g*MA [35]. PE*g*MA is also compatible with the PE matrix due to the internal hydrophobic PE sequences, resulting in enhancing adhesion between the CNF surface and PE matrix.

**Figure 4:**
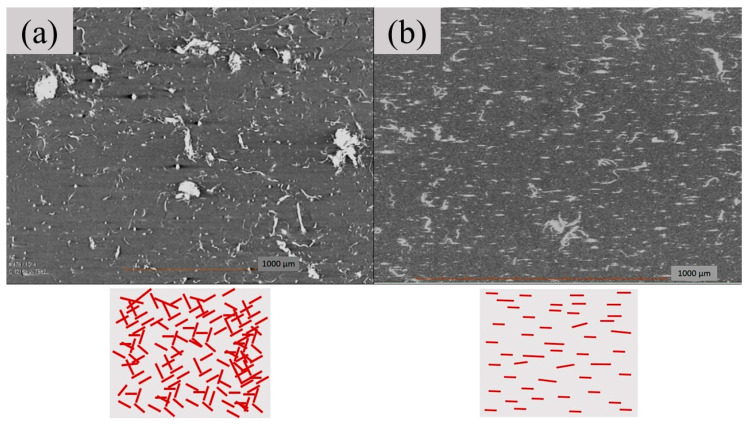
X-CT scan cross-section images of nanocomposites reinforced by 3 wt.% CNF prepared by liquid-assisted (**a**) IMB, and (**b**) TWS. The right illustrations show the distribution and orientation of CNF in the PE matrix.

It is interesting to note that nanocomposites prepared by liquid-assisted TWS recorded higher mechanical properties than nanocomposites prepared by liquid-assisted IMB. The use of liquid-assisted TWS allowed higher concentration of CNF to be dispersed homogeneously in the nanocomposite, which affected mainly the flexural properties of nanocomposites. For instance, flexural strength and modulus of the nanocomposite reinforced by 3% CNF showed the maximum increment of 139% and 195% compared to corresponding nanocomposites prepared by liquid-assisted IMB and PE/PE*g*MA as a reference, respectively. There would be two main reasons, which led to the better mechanical properties of the nanocomposite prepared by TWS: (i) homogeneous dispersion of CNF avoiding agglomeration, and (ii) aligned orientation of CNF in the nanocomposite [36]. To confirm the orientation of CNF in the PE matrix, an X-CT scan of nanocomposites was conducted, and the image is as shown in Figure 4. Although finer CNF portions less than 1 μm were difficult to detect by the X-CT analysis, the observed morphology of the pre-nanofibrillated portions suggests the same morphology of the finer portions.

In reports by Maniruzzaman et al. [37]; Cruz and Viana [26]; Hietala et al. [38]; Salleh et al. [39]; Khanam and AlMaadeed [40], it has been well-understood that the alignment of large size (micro-scale onwards) natural fibers in biocomposites is very much dependent on the melt-processing method. Nevertheless, the effect of processing methods on the alignment of fibers in the polymer matrix has not been studied for nano-scale fibers. Based on the X-CT analysis results in Figure 4, the effect of liquid-assisted TWS processing on CNF alignment is similar to the previous findings using microfiber, in which the liquid-assisted TWS processing has caused the fiber to be well-aligned to the same direction. In the case of liquid-assisted IMB, random alignment and dispersion were observed. Agglomeration of CNF was also occurred, and these have contributed to the lower mechanical properties of the IMB nanocomposites.

The main reason which contributed to the differences between TWS and IMB is the processing mechanism. According to Drobny [41], IMB involves kneading for the preparation of non-continuous homogenous polymer blend. It does not involve pressurized system and extrusion, therefore it contributes to the non-uniform alignment of CNF in the polymer matrix. Due to this, CNF tends to get entangled with each other when they are subjected to the liquid-assisted IMB. Meanwhile, TWS consists of two rotating screws that can continuously melt mixing and dispersive mixing both of the CNF and polymer at high shear force. It is operated in a pressurized system with dies that extrude the nanocomposite strands. The presence of flight around the TWS screw also helps in aligning the CNF. This has caused liquid-assisted TWS process to provide a continuous homogenous polymer blend.

### 3.3. Crystallinity and Thermal Properties of Nanocomposite

The crystallinity index (*CrI*) of PE/PE*g*MA/CNF nanocomposites and PE/PE*g*MA are summarized in Table 4. It was observed that all PE/PE*g*MA/CNF nanocomposites recorded higher *CrI* values than 37.2% for PE/PE*g*MA as reference. For nanocomposites prepared by liquid-assisted IMB, the *CrI* value was approximately between 38.0–43.9%, while for nanocomposites prepared by liquid-assisted TWS, the *CrI* value was evaluated to be around 38.1–44.4%.

These results indicate that the incorporated CNF essentially increased *CrI* of the PE matrix. According to Shalwan and Yousif [42] and Essabir et al. [43], the *CrI* of composite changed by self-nucleation effect (homogenous nucleation) and/or by a nucleating agent (heterogeneous nucleation). Hence, in this case, it is considered that CNF acted as a nucleating agent and produced seed crystals, resulting in the promotion of secondary crystallization and finally increased the *CrI* value of composites [40].

**Figure 5:**
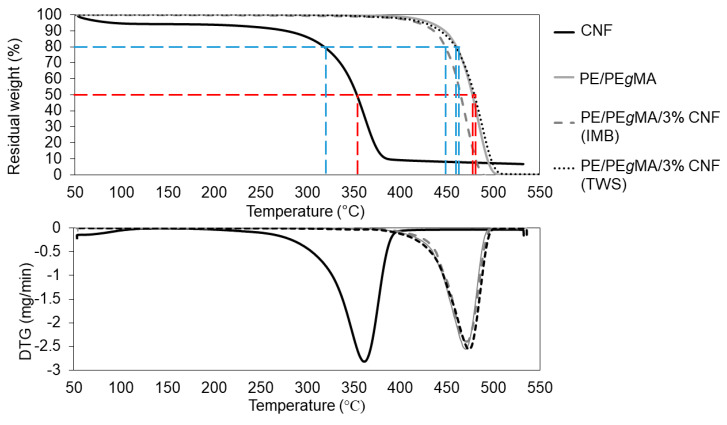
Thermogravimetry (TG) and differential thermogravimetry (DTG) thermograms of CNF, PE/PE*g*MA and PE/PE*g*MA/3% CNF prepared by liquid-assisted IMB and TWS. * The blue line indicates *T*_d20%_ and the red line indicates *T*_d50%_.

The *CrI* results were also in accordance with thermal properties measured by TG and DSC as listed in Table 2. The *T*_d20%_ of the nanocomposites prepared by liquid-assisted IMB was in a range of 444–450 °C, while the *T*_d50%_ was in a range of 464–467 °C, which were lower than 443–459 and 464–478 °C for *T*_d20%_ and *T*_d50%_ of the nanocomposites prepared by TWS, respectively, as shown in Figure 5. It was also noticeable that the highest *T*_d50%_ and *T*_d20%_ values were observed on 0.5% and 3% CNF of PE/PE*g*MA/CNF nanocomposites for liquid-assisted IMB and TWS processing methods, respectively. Similar result of *T*_max_ was also observed for the nanocomposites prepared by these two different methods. In the case of nanocomposites prepared by liquid-assisted TWS, the *T*_max_ was around 473–476 °C, which was higher than 472 °C of PE/PE*g*MA, whereas the nanocomposites prepared by liquid-assisted IMB recorded much lower range, which was around 468–471 °C.

In addition to mechanical properties, the highest thermal properties of PE/PE*g*MA/3% CNF nanocomposite prepared by liquid-assisted TWS processing is also considered to be attributed by homogenous dispersion and less agglomeration of CNF in PE matrix (Figure 3), because the homogeneously dispersed CNF restrained the thermal mobility of PE sequences. On the other hand, agglomerated CNF reduced the thermal properties of composites because of its poorer thermal stability (*T*_d50%_ = 353 °C) than PE.

The crystallization temperature (*T*_c_) and melting temperature (*T*_m_) were determined by DSC measurement. It was revealed that both *T*_c_ and *T*_m_ of PE/PE*g*MA/CNF nanocomposites showed nearly the same values with the neat PE/PE*g*MA. This is attributed by the low CNF loading, in which the amount was insufficient to give effects on the melting behavior of PE/PE*g*MA composites. Similar to *T*_m_*,* the increment of *T*_c_ for all PE/PE*g*MA/CNF nanocomposite is quite low since PE is an elastomeric copolymer [44]. A similar finding has also been reported by Maia et al. [44] that incorporates CNF in the PE matrix and Pollanen et al. [45] that incorporates microcrystalline cellulose (MCC) in the HDPE matrix. Maia et al. [44] revealed that there was no significant differences of *T*_m_ and *T*_c_ between neat PE and PE composites incorporated with CNF in a range of 1, 3, 5, 10, 20 and 30 wt.%, but such differences can be seen in PE incorporated with 90 wt.% CNF composites. In the case of Pollanen et al. [45], the value of *T*_m_ and *T*_c_ remained significant even after 5, 10, 20 and 40 wt.% MCC were added in the HDPE.

By comparing the nanocomposites prepared by different compounding method, the *T*_c_ values of PE/PE*g*MA/CNF nanocomposites prepared by liquid-assisted IMB were slightly higher than the corresponding nanocomposites prepared by liquid-assisted TWS. This suggests that the agglomerates of CNF contributed to the crystallization of PE. On the other hand, the *T*_m_ and *CrI* values increased with increase in CNF content, and both values of PE/PE*g*MA/CNF nanocomposites prepared by liquid-assisted TWS were higher than corresponding nanocomposites prepared by liquid-assisted IMB, suggesting that the more the CNF surface area, the higher the *T*_m_ and *CrI* values showed. Thus, it was recognized that the compounding method of CNF had also clear effects on the thermal properties of the nanocomposites.

## 4. Conclusions

This study demonstrated that CNF can be incorporated in polyolefin such as PE for reinforcement material by liquid feeding of CNF. Results obtained from this study revealed that liquid-assisted TWS contributed to better CNF orientation and dispersion as compared to liquid-assisted IMB. This contributed to significant increment of mechanical properties, up to 195% for flexural strength at CNF composition of 3 wt.%. It was also observed that random and heterogeneous CNF dispersion in nanocomposites led to lower thermal and mechanical properties.

As a conclusion, liquid-assisted TWS is an excellent approach to ensure homogenous dispersion of CNF in the polymer matrix which affected positively on the mechanical, crystallinity and thermal properties of nanocomposites.

## Figures and Tables

**Table 1:** Composition of cellulose nanofiber (CNF), polyethylene (PE) and maleic anhydride-grafted-polyethylene (PE*g*MA) for composite production.

	PE:PE*g*MA (97:3)	
CNF content (wt.%)	PE content (wt.%)	PE*g*MA content (wt.%)
0.5	96.515	2.985
1	96.03	2.97
2	95.06	2.94
3	94.09	2.91
4	93.12	2.88
5	92.15	2.85

**Table 2:** Mechanical properties of PE/PE*g*MA and PE/PE*g*MA/CNF nanocomposites prepared by liquid-assisted IMB and TWS.

Processing Method	CNF (wt.%)	Tensile Strength (MPa)	Young’s Modulus (MPa)	Flexural Strength (MPa)	Flexural Modulus (MPa)
	0 (PE/PE*g*MA)	7.8 ± 0.3 ^Eg^	110 ± 0.01 ^Fe^	5.6 ± 0.5 ^Fg^	219.2 ± 17.6 ^Dg^
**Liquid-assisted internal melt-blending (IMB)**	0.5	9.0 ± 0.2 ^C^	130 ± 0.3 ^C^	9.5 ± 1.2 ^C^	220.7 ± 13.4 ^C^
	1	10.5 ± 0.1 ^A^	145 ± 0.1 ^A^	11.3 ±1.8 ^A^	230.1 ± 12.6 ^A^
	2	10.1 ± 0.4 ^B^	137 ± 1.2 ^B^	10.6 ± 2.6 ^B^	222.0 ± 8.1 ^B^
	3	8.8 ± 0.5 ^D^	125 ± 1.5 ^D^	6.9 ± 2.1 ^D^	139.5 ± 5.5 ^E^
	4	7.3 ± 0.2 ^F^	114 ± 0.01 ^E^	6.0 ± 2.7 ^E^	130.6 ± 10.3 ^F^
	5	6.7 ± 0.3 ^G^	105 ± 0.01 ^G^	5.2 ± 0.8 ^G^	120.1 ± 13.0 ^G^
**Liquid-assisted twin screw extrusion (TWS)**	0.5	9.2 ± 0.7 ^f^	110 ± 0.01 ^e^	8.2 ± 0.3 ^f^	222.7 ± 5.4 ^f^
	1	11.0 ± 0.3 ^c^	141 ± 0.01 ^c^	12.2 ± 2.2 ^d^	245.8 ± 8.6 ^d^
	2	11.5 ± 0.4 ^b^	150 ± 0.02 ^b^	13.7 ± 1.1 ^b^	260.2 ± 7.1 ^b^
	3	12.1 ± 0.1 ^a^	166 ± 0.01 ^a^	16.5 ± 0.2 ^a^	273.0 ± 2.2 ^a^
	4	10.7 ± 0.6 ^d^	138 ± 0.01 ^d^	12.8 ± 1.6 ^c^	250.6 ± 2.7 ^c^
	5	9.3 ± 0.5 ^e^	101 ± 0.01 ^f^	10.5 ± 0.8 ^e^	234.1 ± 3.3 ^e^

All data are means of 5 replicates ± S.D. Capital letters indicate significant difference (*p* < 0.05) among the nanocomposites prepared by liquid-assisted IMB, while small letters indicate significant difference (*p* < 0.05) among the nanocomposite samples prepared by liquid-assisted TWS.

**Table 3:** Mechanical properties of polymer composites incorporated with CNF by liquid feeding method.

Composites	Method of Preparation	Findings (Increment of Mechanical Properties in Comparison to the Neat Polymer, %)		Ref.
PE/PE*g*MA/3% CNF (TWS)	Liquid feeding of PE/PE*g*MA/3% CNF in a twin-screw extruder at 50 rpm with a heating temperature profile of 80, 160, 160 and 160 °C.	Tensile strength	55	This study
		Young’s modulus	51	
		Flexural strength	195	
		Flexural modulus	25	
PE/PE*g*MA/1% CNF (IMB)	Liquid feeding of PE/PE*g*MA/1% CNF in an internal mixer at 50 rpm for 20 min at 160 °C	Tensile strength	35	This study
		Young’s modulus	32	
		Flexural strength	102	
		Flexural modulus	5	
PCL/PMMA-latex/10% CNF nanocomposites	Liquid feeding of PCL/PMMA-latex/10% CNF at 120 °C, at 30 rpm for 5 min and 100 rpm for 10 min	Tensile strength	44	[32]
		Young’s modulus	158	
PCL/20% pulp biocomposite	Liquid feeding of PCL/20% pulp in an extruder at 120 °C, at 30 rpm for 5 min and 100 rpm for 10 min	Young’s modulus	640	[27]
		Yield stress	94	
Cellulose acetate butyrate (CAB)/5% cellulose nanowhiskers (CNW) composites	Liquid feeding of CAB/CNW composites in an extruder at 140–170 °C and the screw speed was 150 rpm.	Tensile strength	95	[33]
		Young’s modulus	300	
PLA/triethyl citrate (TEC)/chitin nanocrystals (ChNC) composites	Liquid feeding of PLA/TEC/ChNC composites in an extruder at 300 rpm at 170–200 °C	Young’s modulus	300	[34]
		Yield strength	478	

**Table 4:** Summary of crystallinity index and thermal properties of PE/PE*g*MA and PE/PE*g*MA/CNF nanocomposites prepared by liquid-assisted IMB and TWS.

		PE/PE*g*MA	PE/PE*g*MA/0.5% CNF	PE/PE*g*MA/1% CNF	PE/PE*g*MA/2% CNF	PE/PE*g*MA/3% CNF	PE/PE*g*MA/4% CNF	PE/PE*g*MA/5% CNF
*T*_d20%_ (°C)	IMB	460 ± 0.10	452 ± 0.10	450 ± 0.08	449 ± 0.11	447 ± 0.09	448 ± 0.11	444 ± 0.13
	TWS		445 ± 0.06	448 ± 0.12	446 ± 0.09	459 ± 0.03	445 ± 0.07	443 ± 0.10
*T*_d50%_ (°C)	IMB	477 ± 0.12	468 ± 0.05	464 ± 0.09	467 ± 0.09	464 ± 0.09	464 ± 0.10	466 ± 0.06
	TWS		465 ± 0.11	466 ± 0.10	466 ± 0.10	478 ± 0.07	464 ± 0.11	465 ± 0.09
*T*_c_ (°C)	IMB	92 ± 0.08	92.3 ± 0.07	92.8 ± 0.09	93.1 ± 0.09	93.3 ± 0.07	93.4 ± 0.10	93.5 ± 0.08
	TWS		92 ± 0.08	92.1 ± 0.06	92.5 ± 0.09	92.4 ± 0.09	92.6 ± 0.09	92.3 ± 0.08
*T*_m_ (°C)	IMB	108.5 ± 0.03	107.8 ± 0.06	107.6 ± 0.06	108.7 ± 0.09	108.0 ± 0.08	107.8 ± 0.07	108.0 ± 0.03
	TWS		108.2 ± 0.05	108.2 ± 0.07	108.4 ± 0.07	108.6 ± 0.05	108.6 ± 0.06	108.8 ± 0.08
*CrI* (%)	IMB	37.2 ± 0.10	38 ± 0.11	38.6 ± 0.09	39.4 ± 0.10	40.1 ± 0.12	41.8 ± 0.09	43.9 ± 0.09
	TWS		38.1 ± 0.12	38.8 ± 0.08	39.7 ± 0.06	40.6 ± 0.11	42.4 ± 0.09	44.4 ± 0.10

Note: All data are means of 3 replicates ± S.D.
